# Matrix Gla Protein Promotes the Bone Formation by Up-Regulating Wnt/β-Catenin Signaling Pathway

**DOI:** 10.3389/fendo.2019.00891

**Published:** 2019-12-20

**Authors:** Jie Zhang, Zhenrong Ma, Kang Yan, Yong Wang, Ya Yang, Xiang Wu

**Affiliations:** ^1^Department of Endocrinology and Metabolism, The Second Attached Hospital of Nanchang University, Nanchang, China; ^2^Department of Parasitology, School of Basic Medical Science, Central South University, Changsha, China; ^3^Department of Endocrinology and Metabolism, Heyuan People's Hospital, Heyuan, China; ^4^Department of Forensic Medicine, School of Basic Medical Science, Central South University, Changsha, China

**Keywords:** MGP, osteoblast, bone formation, Wnt/β-catenin signaling pathway, osteoporosis

## Abstract

**Objective:** Studies suggest that matrix Gla protein (MGP) is associated with osteoporosis. However, the precise mechanism through which MGP regulates bone metabolism is not fully understood. The purpose of this study was to clarify the role of MGP in bone metabolism.

**Methods:** The MGP gene in MG63 cell line was knocked down using shRNA. Cell Counting Kit-8 assay was used to detect the proliferation of MG63 cells. Moreover, the differentiation and mineralization of MG63 cells were measured through alkaline phosphatase staining and Alizarin Red S staining. Western blotting and quantitative reverse transcription-polymerase chain reaction were conducted to detect the protein and mRNA levels of components of the Wnt/β-catenin signaling pathway, such as Wnt3a, β-catenin, and Runx2. Transgenic (MGP+) mice were used to detect the effects of MGP *in vivo*.

**Results:** The Cell Counting Kit-8 assay suggested that upregulated MGP could promote the proliferation of MG63 cells, whereas its downregulation inhibited proliferation. The alkaline phosphatase assay and Alizarin Red S staining showed that overexpressed MGP led to prominently upregulated differentiation and mineralization of MG63 cells. Conversely, knockdown of MGP decreased the levels of differentiation and mineralization. Western blotting and quantitative reverse transcription-polymerase chain reaction showed that overexpression of MGP upregulated Wnt3a, β-catenin, and Runx2. In contrast, knocking down MGP reduced their transcriptional levels. *In vivo*, overexpression of MGP inhibited the decrease in bone mineral density induced via ovariectomy in the femur, and significantly prevented bone volume fraction, trabecular number, BV/TV, and TbTh to decrease. In addition, it increased the levels of estradiol in sera.

**Conclusion:** The findings of this study suggest that the promotion of osteoblast proliferation, differentiation, and mineralization by MGP may be a mechanism to prevent osteoporosis. Furthermore, the results show that MGP promoted the osteogenic effects via the Wnt/β-catenin signaling pathway.

## Introduction

Osteoporosis is a systemic degenerative disease characterized by decreased bone mineral density (BMD) and increased risk of fractures (1, 2). The incidence of this disease increases with advancing age, and has become a global problem for the aging population (2) owing to the potentially devastating results (3) and high cumulative rate of fractures. Matrix Gla protein (MGP) is a secreted protein (12 kDa), widely distributed in the bone, cartilage, heart, and kidney (4). There are various forms of MGP, which differ from one another in their state of phosphorylation and/or carboxylation. Uncarboxylated MGP, one of the inactive forms, was found markedly increased in the foci of vascular calcification (5). Based on available data, uncarboxylated MGP may be a marker of vascular calcification (6, 7). Recently, MGPhas been shown to be an inhibitor of calcification in soft tissues, such as cartilage and vasculature. However, some research studies suggest that MGP may participate in the reconstruction of cancellous bone through the fetuin-mineral-complex, a macromolecular complex existed in serum consisting of calcium, phosphate, fetuin, and MGP (8, 9). We have previously shown that level of MGP in the serum was decreased, whereas uncarboxylated MGP of the lumbar vertebra and arterial endothelium increased in a Sprague-Dawley rat ovariectomy model (10). Moreover, we have observed that low-dose estradiol, vitamin K, parathyroid hormone, and alendronate upregulated the expression of MGP in ovariectomized Sprague-Dawley rats and primary osteoblasts (11). Although previous studies showed that MGP may participate in the process of bone metabolism, the detailed mechanism through which MGP regulates bone formation remains unclear. Therefore, it is worth exploring the possible molecular mechanisms involved in the regulation of osteogenesis by MGP.

The Wnt pathway is another important regulatory pathway in bone formation. Reports showed that Wnt signals regulated bone mass by perpetuating the self-renewal and proliferation of stem cells, inducing differentiation and reducing apoptosis of osteoprogenitor cells (12, 13). Loss-of-function mutation of low-density lipoprotein receptor-related protein 5 was found in patients with osteoporosis (14). The levels of estradiol, low-density lipoprotein receptor-related protein 5, Runx2, and β-catenin were decreased in ovariectomized rats, suggesting that the Wnt/β-catenin signaling pathway may be involved in the pathogenesis of post-menopausal osteoporosis (15). *In vivo* and *in vitro* experiments confirmed that MGP is the main target gene of Runx2, and exogenous Runx2 increased the transcription and expression of MGP (16). Alfieri et al. found that Wnt3a induced MGP expression, whereas secreted frizzled related protein 3, a Wnt inhibitor, blocked the induction of MGP in vascular smooth muscle cells (17). These data suggest that the Wnt signaling pathway may be related to MGP. Therefore, we hypothesized that MGP interacts with the Wnt/β-catenin signaling pathway during osteogenesis; however, the mechanisms involved in this process remain elusive.

In this study, we explored the possible molecular mechanisms involved in the regulation of osteogenesis by MGP. In addition to obtaining new insights into a possible role of MGP in the regulation of osteoblastic activity, we sought to determine whether MGP is involved in Wnt signaling-regulated bone formation.

## Materials and Methods

### Cell Culture

MG63 cells (ATCC, USA) were seeded at 1 × 10^6^ cells/cm^2^ and cultured in Dulbecco's modified Eagle's medium (Gibco, Green island, NY, USA) without phenol red, supplemented with 10% fetal bovine serum (Gibco) and 1% penicillin-streptomycin (Gibco) in a humidified incubator at 37°C under 5% carbon dioxide. The culture medium was changed every 2-3 days. After reaching 80% confluence, cells were rinsed with phosphate-buffered saline (Gibco) and detached using 0.25% pancreatic enzymes containing 0.02% ethylenediaminetetraacetic acid for 1-2 min. Complete culture medium was added to inactivate the enzymes. The cells were counted and subcultured at a ratio of 1:3.

### Cell Transfection

MG63 cells were seeded in six-well plates at a density of 1 × 10^6^/ml, and the cells reached 80% confluency the following day. The transfections were performed using 4 μg pIRES2-enhanced green fluorescent protein-MGP (pIRES2-EGFP-MGP) (overexpression group), pIRES2-EGFP (vehicle control), pKLO-MGP-shRNA (knockdown group), or pKLO-EGFP (vehicle control) in 250 μl serum-free Dulbecco's modified Eagle's medium after prior incubation with the transfection reagent transfectamin 2000 (Invitrogen, USA) at room temperature for 10 min. The medium was changed 4-6 h later.

### Cell Proliferation Assay

Cell Counting Kit-8 assay was used to determine the proliferation of cells. MG63 cells were seeded in a 96-well plate at a density of 1 × 10^4^/ml after transfection for 24 h, and incubated with growth medium. After culture for 24, 48, 72, and 96 h, the cells were treated with Cell Counting Kit-8 reagent (DOJINDO, Tokyo, Japan) for 2 h. The optical density was monitored using a multiscan spectrum at a wavelength of 450 nm to construct growth curves.

### Alkaline Phosphatase (ALP) Staining and Activity Assay

The transfected MG63 cells were seeded at a density of 1 × 10^5^/cm^2^ in a 12-well plate, and cultured in osteogenic induction medium consisting of growth medium supplemented with 10 mM β-glycerophosphate (Sigma-Aldrich, St. Louis, MO, USA), 0.1 mM dexamethasone (Sigma-Aldrich), and 50 mM ascorbic acid (Sigma-Aldrich). Cells were cultured for 7 consecutive days. For the ALP staining assay, the ALP Double-Stain kit (Beijing Leagene Biotechnology CO. Ltd., Beijing, China) was used according to the instructions provided by the manufacturer. Images were visualized using a bright-field optical microscope. The ALP activity was detected using an ALP kit (Nanjing built Technology Co. Ltd., Nanjing, China) according to the protocol provided by the manufacturer.

### Alizarin Red S Staining

The transfected MG63 cells were seeded at a density of 1 × 10^5^/ml in a six-well plate, and cells were cultured in osteogenic induction medium. The medium was changed every 2 days. The mineralized nodules were visualized using an Alizarin Red-Stain kit (Beijing Leagene Biotechnology Co. Ltd.) according to the instructions provided by the manufacturer. Images were visualized using a bright-field optical microscope. For quantification, Alizarin Red was solubilized with 10% hexadecylpyridinium chloride (Tokyo Chemical Industry Co. Ltd., Tokyo, Japan), followed by incubation at room temperature for 1 h. The quantity of Alizarin Red was measured using a multiscan spectrum at a wavelength of 562 nm.

### Real-Time Quantitative Reverse Transcription-Polymerase Chain Reaction

Total RNA was extracted using the Trizol reagent (Takara Japan Bio Co. Ltd., Japan) and reverse-transcribed using the PrimeScript RTMaster Mix (Takara Japan Bio Co. Ltd.). The primer sequences are listed in Supplementary Table 1. The mRNA expression of housekeeping gene glyceraldehyde-3-phosphate dehydrogenase, as well as that of target genes encoding MGP, Wnt3a, β-catenin, and Runx2 was determined using the SYBR Premix Ex Taq II (Takara Japan Bio Co. Ltd.) on an Applied Biosystems 7500 machine (Thermo Fisher Scientific, Waltham, MA, USA). The polymerase chain reaction conditions were as follows: 95°C for 30 s for stage 1; 40 cycles of 95°C for 5 s and 64°C for 34 s for stage 2. The expression of target genes was normalized to that of glyceraldehyde-3-phosphate dehydrogenase. The relative expression was calculated using the formula 2^−ΔΔCt^.

### Western Blotting

Western blotting was performed routinely. In brief, after cell lysis, the protein concentration in the lysate was determined using a bicinchoninic acid protein assay kit (Thermo Fisher Scientific). Total protein (30 μg per sample) was run on 15 and 8% sodium dodecyl sulfate-polyacrylamide gel electrophoresis gels, followed by the wet-transfer process using polyvinylidene difluoride membranes (Merck Millipore, Germany). Subsequently, the reaction was blocked with 5% skim milk powder at room temperature for 1 h. Immunoblotting analysis was performed through incubation with primary antibodies against MGP (Abcam, Cambridge, UK), Wnt3a (Abcam), β-catenin (Abcam), Runx2 (Abcam), and β-actin (ZSGB-Bio, Beijing, China) overnight on a shaking table at 4°C. The samples were subsequently incubated for 1 h at room temperature with horseradish peroxidase-conjugated anti-rabbit or anti-mouse secondary antibodies (Santa Cruz Biotechnology, Santa Cruz, CA, USA). The results were normalized to the loading control β-actin, and an enhanced chemiluminescence detection system was used for the data analysis.

### Transgenic (MGP+) Mouse Model Animals, Treatments, and Specimen Collection

The study protocol was approved by Nanjing University-Nanjing Institute of Biomedical Sciences. Specific-pathogen-free, virgin, female, Sprague-Dawley mice (age: 3 months; weight: 180 ± 12.0 g) (*n* = 60) were housed in an air-conditioned room (22°C) under a 12-h light/dark cycle. The mice were placed on a rack, in multiple standard rodent cages (545 × 395 × 200 mm). Ethical approval for the use of animals in this study was granted by the Animal Research Ethics Committee of China. During the experimental period, all mice were provided with distilled water and standard mouse chow *ad libitum* on a daily basis. The animals were monitored through observation of the stable baseline and circadian variation in temperature and activity daily. Acclimatized mice underwent either bilateral laparotomy (sham, *N* = 10) or bilateral ovariectomy (OVX; *N* = 50) after being anesthetized with 3% sodium pentobarbital (50 mg/kg) via an intraperitoneal injection. After the surgical procedures, monitoring of the animals during the recovery period assists in confirming surgical recovery. Notably, sham mice were fed in parallel with the OVX mice to minimize differences in body weight between the two surgical groups. Four weeks after surgery, the mice were randomly divided into four treatment groups (*N* = 20 per group): a sham-operated group, an OVX group, a transgenic (MGP+) sham-operated group, and a transgenic (MGP+) OVX group. The body weights of the animals were recorded weekly during the experimental period. One day prior to euthanasia, each mouse was housed individually without food for 24 h in a metabolic cage. A urine sample was collected from each mouse, and the urine was acidified with 2 ml of 1 Mm hydrochloric acid. On the following day, the mice were anesthetized with 3% sodium pentobarbital (50 mg/kg) via an intraperitoneal injection and sacrificed. A blood sample was collected via abdominal aorta puncture, and serum was prepared through centrifugation (2,000 rpm for 20 min). Urine and serum samples were stored at −80°C for biochemical analysis. Femurs were dissected and stored in normal saline at −20°C for bone analyses.

### Assessment of BMD and Bone Microarchitecture

Two-dimensional total bone mineral content and total BMD in the right femur of each mouse were measured using dual-energy X-ray absorptiometry with the Lunar Prodigy Advance System (GE Healthcare, USA). The system was equipped with appropriate software for the assessment of bone density in small animal's laboratory. The BMD was calculated using the bone mineral content of the measured area and reported as g/cm^3^.

After measurement of the BMD, we selected three representative right femurs from each group to evaluate the trabecular micro-architecture of the femoral metaphysis through micro-computed tomography (μCT; Scanco Medical, Zurich, Switzerland). The selection of representative samples was based on the median value of the total BMD of the respective group. The trabecular bone is rich in the distal femur compared with the proximal and middle regions; thus, the scan was performed from the proximal growth plate in the distal direction (16 μm per slice) for each selected femur sample. A total of 350 images were captured from this region of each femur using a 1,024 × 1,024 matrix, resulting in an isotropic voxel resolution of 22 μm^3^.

The volume of interest was selected as a cross-sectional area spanning 25-125 slices from the proximal growth plate. The scanning generated three-dimensional images of the micro-architecture were examined and displayed. Bone morphometric parameters obtained from the μCT, including the bone volume fraction, trabecular number, trabecular separation, trabecular thickness, connectivity density, and structure model index, were analyzed for the volume of interest. The operator conducting the μCT analysis was blinded to the treatments. All examinations were performed according to the principles and procedures described in the most recent National Research Council publication of the Guide for the Care and Use of Laboratory Animals and refer to the ARRIVE guidelines.

### Serum

An enzyme-linked immunosorbent assay kit (Biomedical Technologies, Stoughton, MA, USA), with 4% intra- and 7% inter-assay variabilities, was used according to the instructions provided by the manufacturer.

### Statistical Analysis

Data were presented as mean ± standard deviation and analyzed using Student's *t*-test or analysis of variance with the SPSS Statistics Version 20 software (IBM Corp., Armonk, NY, USA). *P* < 0.05 denoted statistical significance.

## Results

### Exogenous MGP Accelerated the Proliferation of MG63 Cells

**Figure 1:**
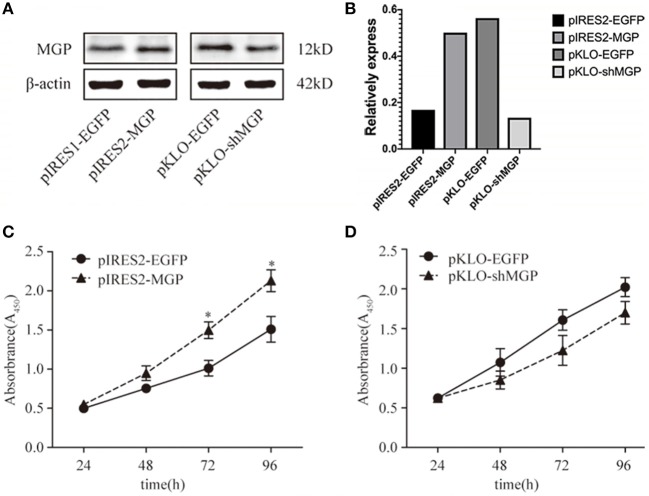
Exogenous MGP accelerated the proliferation of MG63 cells. **(A)** Western blotting results of MGP after transfection. The protein expression of MGP was increased after transfection with pIRES2-MGP vs. the control transfected with pIRES2-EGFP. The expression of MGP was significantly decreased after transfection with pKLO-MGP-shRNA (pKLO-shMGP) vs. the control transfected with pKLO-EGFP. **(B)** The denstometric analysis of western bloting. **(C,D)** The proliferation of MG63 cells was evaluated using the CCK-8 assay after overexpression or downregulation of MGP. The ODs of 4 consecutive days were determined to construct a growth curve. Overexpression of MGP (pIRES2-MGP) facilitated the proliferation of MG63 cells (*P < 0.05). However, there was no statistical significance between the MGP knockdown group (pKLO- shMGP) and control group (pKLO-EGFP) (*P < 0.05).

Proliferation is one of the most important indicators of the osteogenic effect. Therefore, we observed the effect of MGP on the proliferation of MG63 cells. The results of the western blotting analysis of the four groups of transfected MG63 cells and the corresponding cell growth curves are shown in Figure 1. We found that MG63 cells in the MGP overexpression group exhibited accelerated proliferation at 72 and 96 h compared with control (*P* < 0.05, Figure 1C). However, knockdown of MGP using pKLO-MGP-shRNA did not have a significant effect on the proliferation activity of MG63 cells (*P* > 0.05) (Figure 1D). These results indicate that MGP regulated the proliferation of MG63 cells, and exogenous MGP promoted the proliferation of osteoblast MG63 cells.

### Overexpression of MGP Enhanced Osteogenic Differentiation, Whereas Its Downregulation Inhibited Differentiation

**Figure 2:**
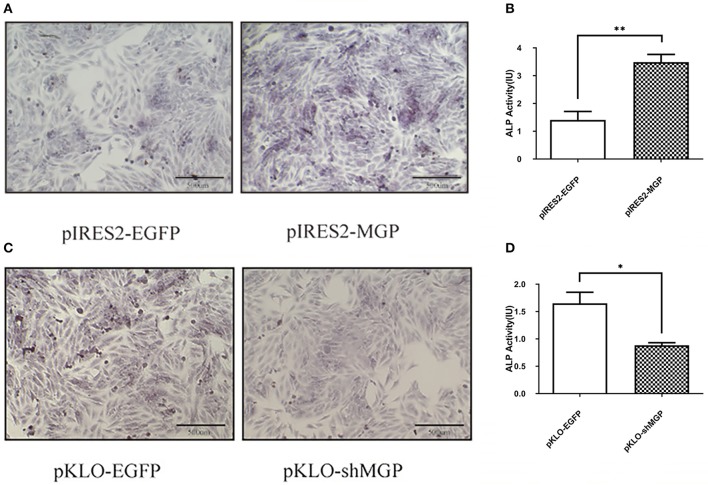
Overexpression of MGP enhanced osteogenic differentiation, whereas its downregulation inhibited differentiation. **(A,C)** Alkaline phosphatase (ALP) staining (Azo coupling method) of MG63 cells after induction of mineralization for 7 days; the ALP activation parts appeared blue. **(B,D)** ALP activity of MG63 cells; both ALP staining and ALP activity showed an increase after overexpression of MGP (pIRES2-MGP) compared with the negative control (pIRES2-EGFP). ALP activity was reduced after knockdown of MGP (*P < 0.05, **P < 0.01).

We subsequently investigated the effect of MGP on the differentiation of MG63 cells. Differentiation was assessed through ALP staining and detection of ALP activity. ALP staining showed significant enhancement in the overexpression group vs. the control group (Figures 2A,B). Moreover, the results of the ALP activity assay were consistent with those obtained through ALP staining. The ALP activity in the overexpression group demonstrated an ~3-fold increase vs. the control group In contrast, the ALP activity in the knockdown group showed an ~2-fold reduction vs. control (*P* < 0.05, Figures 2C,D). These results suggest that overexpression of MGP may promote osteogenic differentiation, whereas its downregulation inhibited differentiation.

### MGP Promoted Osteoblast Mineralization

**Figure 3:**
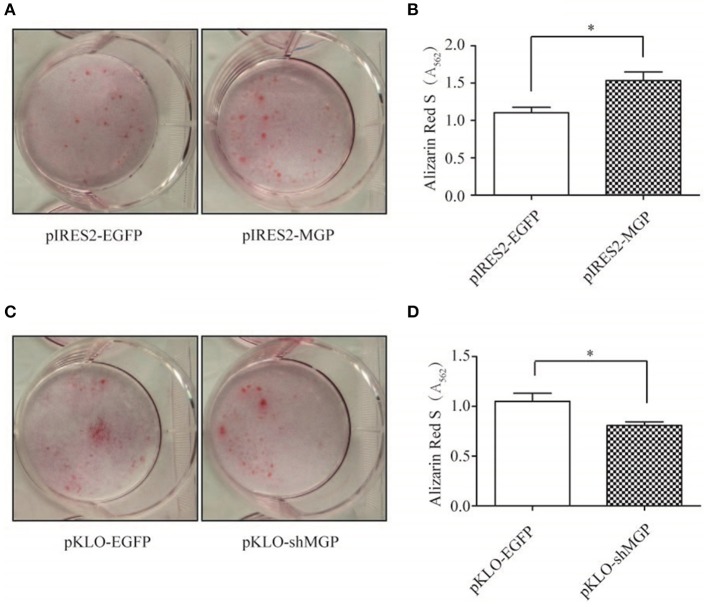
MGP promoted osteoblast mineralization. **(A,C)** Calcium deposits were detected using alizarin red S staining. Mineralization of MG63 cells was induced for 14 days, and the calcium deposits appeared red. **(B,D)** Semi-quantitative analysis of the calcium deposits using cetylpyridinium chloride. MG63 cells transfected with pIRES2-MGP showed significantly higher degree of mineralization than the pIRES2-EGFP group, whereas a markedly lower degree of mineralization was observed in the pKLO-shMGP group. The data are representative of three independent experiments (*P < 0.05).

Furthermore, we examined the mineralization of MG63 through Alizarin Red staining, and quantified the amount of calcium deposition by solubilizing Alizarin Red with 10% hexadecylpyridinium chloride. The intensity of Alizarin Red staining was significantly increased after overexpression of MGP (Figure 3A), and the amount of calcium deposition showed an ~1.5-fold increase in the overexpression group vs. the control group (*P* < 0.05, Figure 3B). In contrast, downregulation of MGP inhibited the mineralization of osteoblasts (Figures 3C,D).

### MGP Promoted the Osteogenic Effects via the Wnt/β-Catenin Signaling Pathway

**Figure 4:**
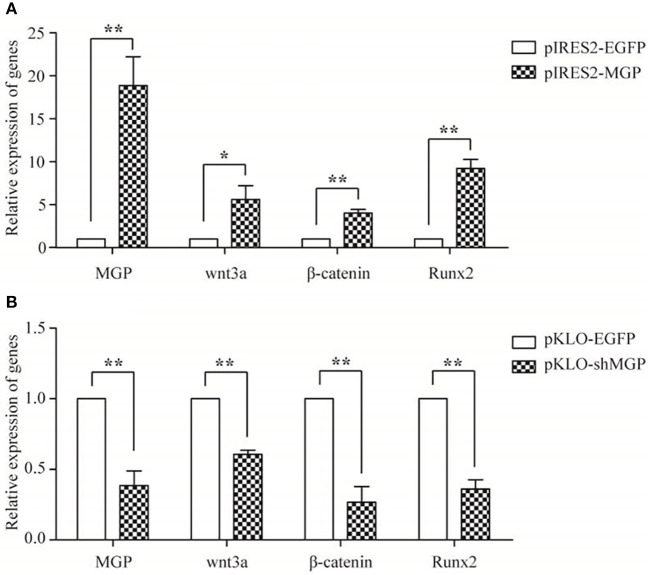
The mRNA expression of MGP and Wnt-related factors. MGP, Wnt3a, β-catenin, and Runx2 were analyzed using real-time RT-PCR after transfection for 48 h. **(A)** The expression of Wnt3a, β-catenin, and Runx2 mRNA was markedly upregulated in the pIRES2-MGP group vs. the pIRES2-EGFP group. **(B)** On the contrary, the pKLO- shMGP group showed a significant decrease in the mRNA levels of Wnt3a, β-catenin, and Runx2 (*P < 0.05, **P < 0.01).

**Figure 5:**
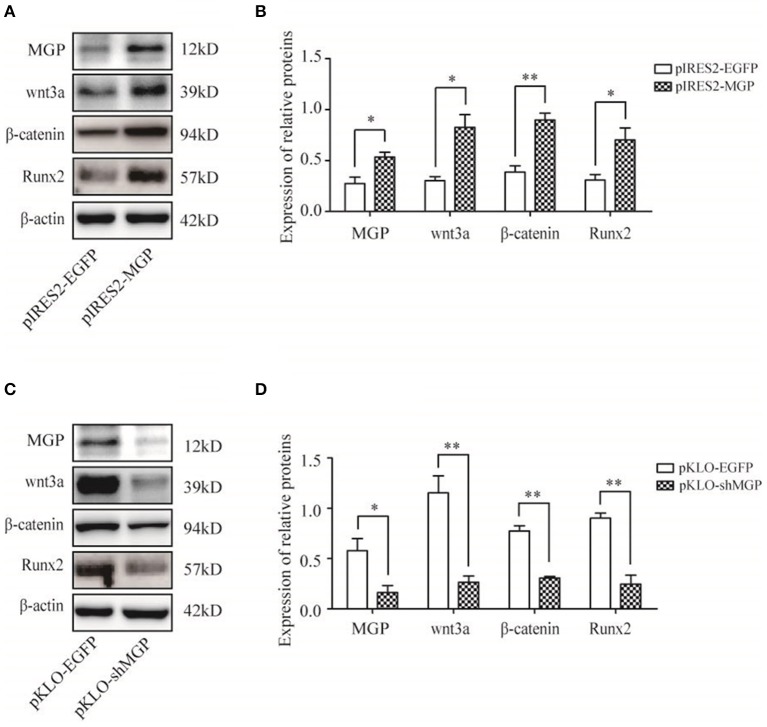
Protein expression of MGP and Wnt-related factors. **(A,C)** MGP, Wnt3a, β-catenin, and Runx2 protein were assessed through western blotting analysis after transfection for 3 days. **(B,D)** The results were consistent with the mRNA expression data (*P < 0.05, **P < 0.01).

We measured the expression levels of the major elements in the Wnt signaling pathway after overexpression or knockdown MGP in MG-63 cells to further elucidate their interaction in osteogenesis. As shown in Figure 4, the mRNA expression levels of the three important factors of the Wnt/β-catenin pathway, namely Wnt3a, β-catenin, and Runx2, showed an 5.94-, 4.03-, and 9.23-fold increase, respectively, in the overexpression group vs. the control group (^*^P < 0.05, ^**^P < 0.01, Figure 4A). Of note, knockdown of MGP suppressed the expression of those three genes. Moreover, in the knockdown group, Wnt3a, β-catenin, and Runx2 demonstrated a 0.61-, 0.26-, and 0.36-fold decrease compared with the control group (^*^P < 0.05, ^**^P < 0.01, Figure 4B). The results of western blotting were consistent with those of the quantitative polymerase chain reaction. Overexpression of MGP enhanced the protein levels of Wnt3a, β-catenin, and Runx2 (2.73-, 2.32-, and 2.27-fold increase, respectively) vs. those observed in the control group (*P* < 0.05, Figures 5A,B). On the contrary, the expression of those related factors in the knockdown group were decreased (*P* < 0.05) (Figures 5C,D). These results suggested that MGP is the target of the Wnt/β-catenin signaling pathway and a positive regulator of Wnt signaling.

### Body Weight in Transgenic (MGP+) Mouse Model Animals

**Figure 6:**
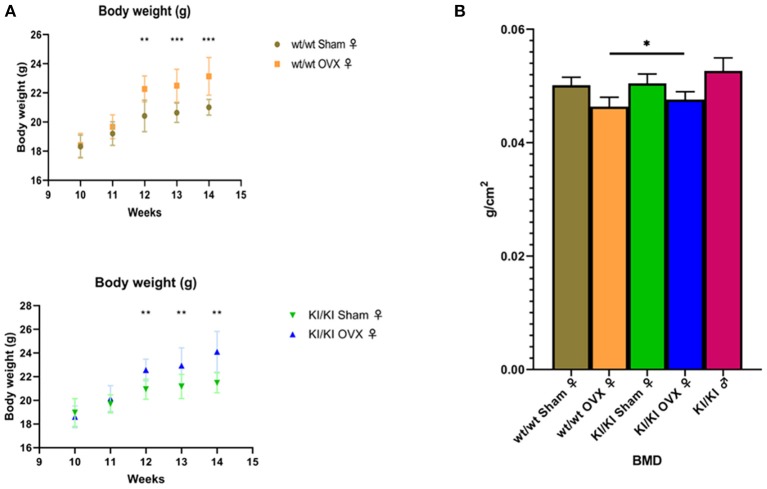
The body weight of the non-transgenic female mice in the sham operation group (wt/wt sham) at week 12 was significantly lower than that of the non-transgenic female mice in the ovariectomized group (wt/wt OVX) (*P < 0.05). Similarly, the body weight of the transgenic female mice in the sham operation group (KI/KI sham) was significantly lower than that measured in the ovariectomized group (KI/KI OVX) (*P < 0.05, **P < 0.01, ***P < 0.001) **(A)**. The BMD of KI/KI OVX mice was significantly higher than that of wt/wt OVX (*P < 0.05) **(B)**.

All mice in the four groups had similar starting weight, were given the same feed, and fed in the same environment. The body weight of the non-transgenic female mice in the sham operation group (wt/wt sham) at week 12 was significantly lower than that of the non-transgenic female mice in the ovariectomized group (wt/wt OVX) (*P* < 0.05). Similarly, the body weight of transgenic female mice in the sham operation group (KI/KI sham) was significantly lower than that reported in the ovariectomized group (KI/KI OVX) at week 12 (*P* < 0.05) (Figure 6A).

### BMD in Transgenic (MGP+) Mouse Model Animals

The BMD was calculated using the bone mineral content of the measured area and reported as g/cm^3^. The mean bone density (BMD) of KI/KI OVX mice, KI/KI sham and wt/wt sham mice was significantly higher than that of wt/wt OVX (^*^p < 0.05), while the BMD of wt/wt sham and KI/KI sham mice was not significantly different from that of wt/wt sham mice. The results illustrated that overexpression of MGP inhibited the decrease in the BMD induced by OVX in the femur (*P* < 0.05) (Figure 6B).

### Estradiol Level Assay in Sera

**Figure 7:**
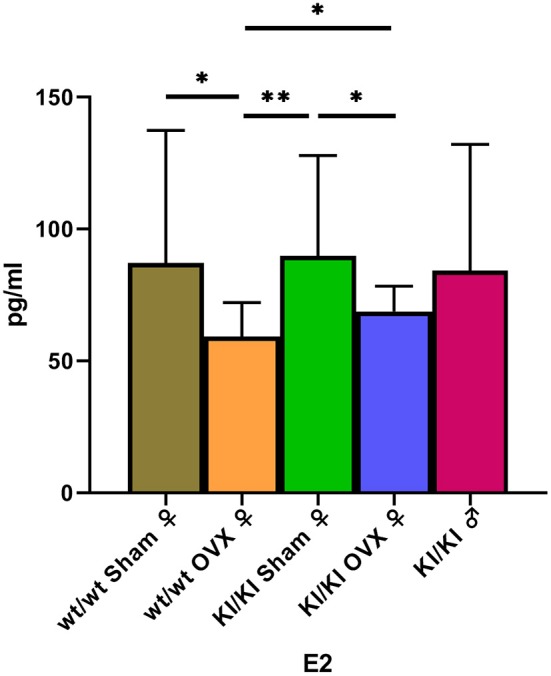
The levels of Estradiol in KI/KI OVX were significantly higher than those recorded in wt/wt OVX mice (*P < 0.05, **P < 0.01).

The levels of Estradiol in the serum were detected using the enzyme-linked immunosorbent assay. The levels of E2 in KI/KI OVX, KI/KI sham, and wt/wt sham mice were significantly higher than those measured in wt/wt OVX mice (^*^P < 0.05) (Figure 7).

### Mic-CT Result

**Figure 8:**
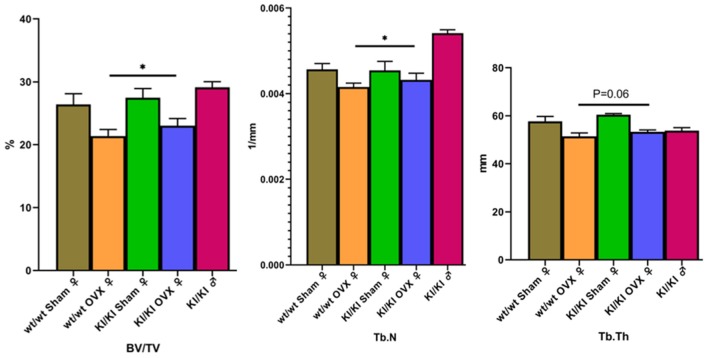
The results show that KI/KI OVX mice, BV/TV, and Tb.N were significantly increased compared with wt/wt OVX mice (*P < 0.05). Overexpression of MGP promoted the levels of bone TbTh (*P* = 0.06) to decrease.

Mic-CT was used to detect trabecular number (TbN) and BV/TV for the evaluation of bone microstructure. The results showed that overexpression of MGP significantly promoted the levels of bone trabecular number and BV/TV (*P* < 0.05) to decrease. Overexpression of MGP promoted the levels of bone TbTh (*P* = 0.06) to decrease (Figure 8).

## Discussion

In the present study, we demonstrated a positive role of MGP in osteoblast proliferation, differentiation, and osteogenesis *in vitro*. The results suggest that overexpression of MGP promotes the proliferation of osteoblasts. Furthermore, overexpression of MGP increased the differentiation and osteogenic capability of MG63 cells, whereas its knockdown inhibited the process of osteoblast differentiation and osteogenic capability of these cells. The mechanism assay confirmed that MGP regulated the functions of osteoblast through the Wnt/β-catenin signaling pathways. These data suggest a potential important role of MGP in the regulation of bone formation.

Bone mineralization is an essential process in improving skeletal hardness and strength. Osteoblasts pass through a series of stages prior to reaching maturity, including cell proliferation, matrix maturation, and matrix mineralization (18). In our study, the proliferation of MG63 cells was facilitated after overexpression of MGP. Bostrom et al. showed that proliferation of bovine aortic endothelial cells was significantly increased after treatment with human-MGP or transfected MGP (19). In addition, Cario-Toumaniantz et al. assumed that high expression of MGP in varicose veins may contribute to venous wall remodeling by improving the proliferation and mineralization processes (20). However, another study found that MGP inhibited the proliferation of vascular mesenchymal cells and smooth muscle cells (21). The data suggest that the influences of MGP on cell proliferation may vary between cell types. In the process of bone formation, ALP activity and the level of mineralization are important markers of osteoblast differentiation and mineralization (22). In the present study, after pIRES2-MGP transfection, ALP staining indicated an upregulated osteoblast phenotype, and Alizarin Red S staining confirmed an induction of mineralization in MG63 cells. In contrast, transfection with the pKLO-MGP-shRNA reversed the aforementioned effects. Li et al. demonstrated that MGP contributes to the osteogenic differentiation and mineralization of human periodontal ligament cells via BMP-2 (23).

Recently, Yao et al. found that loss of MGP causes dysregulation of early endothelial differentiation (24). Similarly, our results indicated that MGP promoted differentiation and mineralization in osteoblast-like cells. However, overexpression of MGP in human trabecular meshwork cells showed a reduction in ALP activity (25). Moreover, Marulanda et al. showed that both differentiation and mineralization were inhibited through transgenic overexpression of MGP in vascular smooth muscle cells (26). Interestingly, another study found that MGP was expressed in satellite cells at high levels during both proliferation and differentiation. Interestingly, a reduction in the expression of MGP decreased proliferation, yet increased differentiation (27). This evidence suggests that MGP may play different roles in various cells and tissues. We hypothesized that MGP acts as an inhibitor in soft tissues and promotes bone formation in osteogenic lineages. Dan et al. noted that expression of MGP in the avian growth plate is timely and spatially regulated, for which MGP is specifically expressed prior to the initiation of ossification in healthy and impaired growth plates (28). This may, to some degree, support our hypotheses. The present results show that MGP promoted bone regeneration by accelerating the proliferation and differentiation of osteoblasts. *In vivo*, we demonstrated that MGP can prevent estrogen deficiency-induced bone loss and deterioration of trabecular micro-architecture in adult OVX mice, thereby maintaining the biomechanical competence of bone. MGP exerts these positive effects on bone remodeling by stimulating the proliferation of osteoblasts. The HE staining results showed that the trabecular bone was obviously rare, thinner and wider, with uneven trabecular break point in wt/wt OVX mice (Supplementary Figure 1).

The transcription factor Runx2 is a crucial early marker of osteogenesis. Runx2 is essential for the differentiation of osteoblasts at an early stage. Mice with a homozygous mutation in Runx2 showed complete absence of bone formation and expired immediately after birth (29). Ducy et al. found that Runx2 regulates the differentiation of osteoblasts by regulating the expression of osteoblast marker genes in osteoblasts and non-osteoblastic cells, such as osteocalcin, type 1 collagen, bone sialoprotein, and osteopontin (30). In our study, overexpression of MGP effectively increased the mRNA and protein levels of Runx2, whereas its knockdown downregulated the expression of Runx2. Considering that overexpression of MGP promoted osteoblast differentiation, we assumed that MGP regulated the expression of Runx2 to control this process. However, several studies have indicated that MGP is a major target gene of Runx2, based on a significant upregulation of MGP after overexpression of Runx2 (31). Therefore, we speculated there may be a feedback control loop between MGP and Runx2, which requires further study.

The Wnt/β-catenin pathway plays important roles in skeletal homeostasis and bone repair. Studies found that the gain-of-function mutations of Wnt in humans caused high bone mass phenotypes, such as van Buchem disease. In contrast, loss-of-function mutations resulted in osteopenic diseases, such as osteoporosis pseudoglioma syndrome (32). These data suggest that modulation of the Wnt signaling pathway is a viable path for improving osteogenesis. Moreover, β-catenin stem cells, inducing osteoblast formation, reduce the apoptosis of mature osteoblasts through direct stimulation of Runx2 gene expression (13, 33, 34). In the current study, we found that overexpression of MGP activated the Wnt/β-catenin pathway by increasing the mRNA and protein levels of Wnt3a, β-catenin, and Runx2. In contrast, knockdown of MGP suppressed their expression. Previous studies indicated that Wnt3a enhanced the proliferation of mesenchymal progenitor cells and osteoblasts (35, 36), while β-catenin promoted osteoblast differentiation, maturation, and mineralization (37, 38). We found MGP potentiated the wnt3a-induced express of early and later osteogenic makers, such as alkaline phosphatase (ALP) in Figure 2 as well as matrix mineralization (Figure 3). Mechanistically, MGP up-regulated wnt3a to induce β-catenin translocation from cell-cell contacts into the cytosol and nucleus, thereby activating wnt/β-catenin signaling. The present study showed that overexpression of MGP accelerated proliferation, differentiation, and mineralization of osteoblasts. We assumed that MGP may promote skeletal anabolic activities through a Wnt/β-catenin signaling-associated mechanism. MGP upregulated the expression of Wnt3a and β-catenin, enhanced cell proliferation, differentiation, and mineralization, and consequently promoted osteogenesis.

In conclusion, the data obtained in this study indicate that MGP is a novel positive regulator of osteoblast proliferation, differentiation, and osteogenesis through interaction with the Wnt/β-catenin signaling pathway *in vitro*. Therefore, these results provide new insights into the possible use of MGP as a therapeutic target in the management of osteoporosis. Although our *in vitro* results supported the role for MGP in modulating bone formation, further investigations are warranted to verify these findings in an intact tissue and *in vivo* setting.

## Data Availability Statement

The datasets generated for this study are available on request to the corresponding author.

## Author Contributions

XW and YY made a conceptualization for the study. ZM and JZ designed the study. The study and the experiments were performed by ZM, JZ, KY, and YW. YW analyzed the data. KY drew the graphs. ZM and JZ wrote the original draft preparation. XW and YY reviewed and determined the final version.

### Conflict of Interest

The authors declare that the research was conducted in the absence of any commercial or financial relationships that could be construed as a potential conflict of interest.

## Supplementary Material

Supplementary Table 1 Sequences of primers.

Supplementary Figure 1 The H&E staining of lumbar vertebral bone structure of **(A)** wt/wt sham; **(B)** wt/wt OVX; **(C)** KI/KI sham; **(D)** KI/KI OVX. And the mice of KI/KI OVX, KI/KI sham, and wt/wt sham showed uniform and dense bone trabeculae. However, in wt/wt OVX mice, trabecular bone was obviously rare, thinner and wider, with uneven thickness and trabecular break point was often observed.
