# Supplementary material for: Matrix Gla Protein Promotes the Bone Formation by Up-Regulating Wnt/β-Catenin Signaling Pathway
Source: Front Endocrinol (Lausanne). 2019 Dec 20;10:891. doi: 10.3389/fendo.2019.00891 (PMC6933527; doi:10.3389/fendo.2019.00891)
Supplement: Supplementary Table 1 — Sequences of primers. [file Table_1.DOCX]

Supplementary Table 1. Sequences of primers

| Targeted gene | Forward primer（5’-3’） | Reverse primer（5’-3’） |
| --- | --- | --- |
| MGP | GATCCTTCTTGCCATCCTG | GTAGTCATCACAGGCTTCC |
| wnt3a | GAGATGGTGGTGGAGAAG | ACCAGTGGAACACGGCAG |
| β-catenin | TTTGCGTGAGCAGGGTGCCA | TTGCTGCTGTGTCCCACCCA |
| Runx2 | CCACAAGGACAGAGTCAG | CTGCCTGGCTCTTCTTAC |
| GAPDH | GAGGGCTGCTTTTAACTCTGGT | GATTTTGGAGGGATCTCGCT |
