# Supplementary figures and images for: Matrix Gla Protein Promotes the Bone Formation by Up-Regulating Wnt/β-Catenin Signaling Pathway
Source: Front Endocrinol (Lausanne). 2019 Dec 20;10:891. doi: 10.3389/fendo.2019.00891 (PMC6933527; doi:10.3389/fendo.2019.00891)

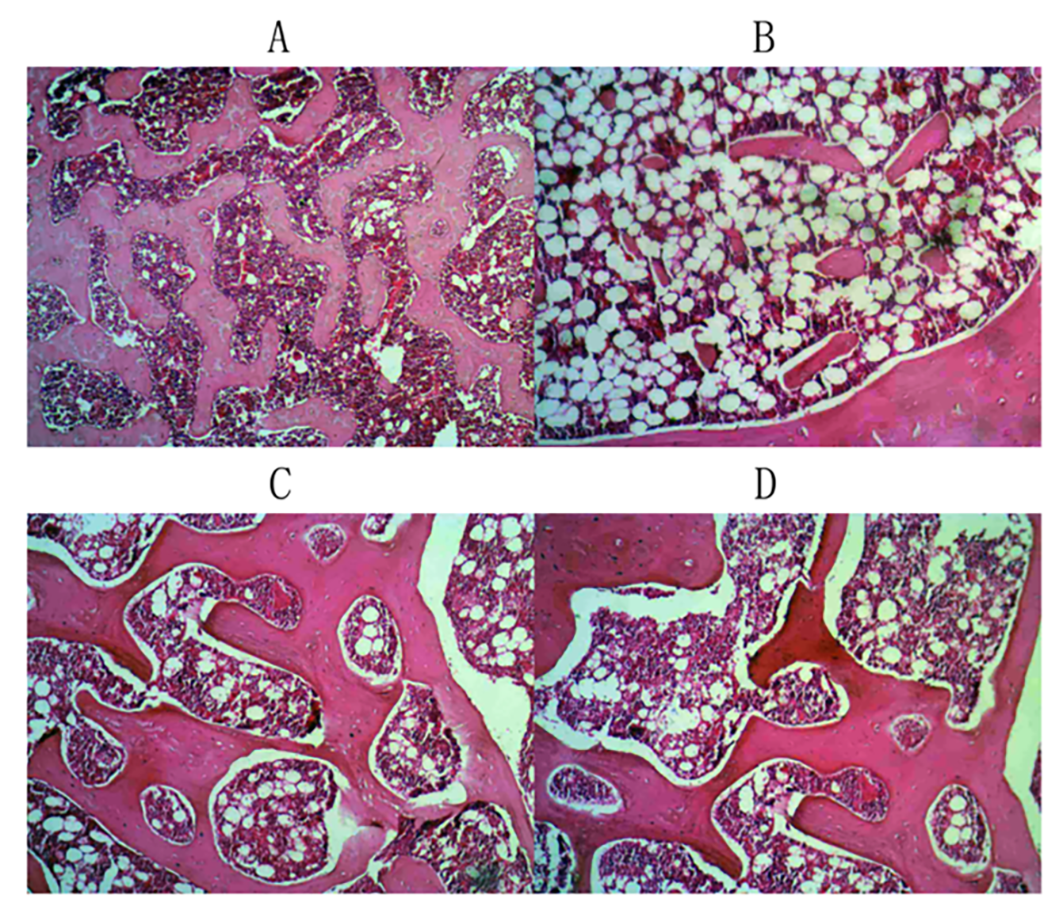

Supplement: Supplementary Figure 1 — The H&E staining of lumbar vertebral bone structure of (A) wt/wt sham; (B) wt/wt OVX; (C) KI/KI sham; (D) KI/KI OVX. And the mice of KI/KI OVX, KI/KI sham, and wt/wt sham showed uniform and dense bone trabeculae. However, in wt/wt OVX mice, trabecular bone was obviously rare, thinner and wider, with uneven thickness and trabecular break point was often observed. [file Image_1.TIF]
